# Dr. Fletcher D. Mooney

**Published:** 1897-12

**Authors:** 


Dr. Fletcher D. Mooney, of St. Louis, died at his home in that city, Monday, November 8, 1897, of Bright's disease, aged 41 years. Dr. Mooney was a man of marked ability, of sterling worth and one who had accomplished more than is usual at his period of life. He graduated at the Missouri medical college in 1878, and has been for many years active in teaching, in medical society work and in the practice of his profession. He was a Fellow of the American Association of Obstetricians and Gynecologists, in which organisation he gave promise of taking a foremost place. His death, though apparently sudden, has been feared by his family and most intimate friends for many months.

